# The heritability and patterns of DNA methylation in normal human colorectum

**DOI:** 10.1093/hmg/ddw072

**Published:** 2016-03-02

**Authors:** Amy Rowlatt, Gustavo Hernández-Suárez, María Carolina Sanabria-Salas, Martha Serrano-López, Konrad Rawlik, Eva Hernandez-Illan, Cristina Alenda, Adela Castillejo, Jose Luis Soto, Chris S. Haley, Albert Tenesa

**Affiliations:** 1 ^1^The Roslin Institute, The University of Edinburgh, Edinburgh, EH25 9RG, UK,; 2 ^2^Grupo de Investigación Epidemiológica and; 3 ^3^Grupo de Investigación en Biología del Cáncer, Instituto Nacional de Cancerología, Bogotá, Colombia,; 4 ^4^Departamento de Química, Universidad Nacional de Colombia, Bogotá, Colombia,; 5 ^5^Laboratorio Genética Molecular, Hospital General Universitario de Elche, 03203 Elche, Alicante, Spain,; 6 ^6^Pathology, Alicante University Hospital, Alicante, Spain and; 7 ^7^MRC Human Genetics Unit, MRC Institute of Genetics and Molecular Medicine, The University of Edinburgh, Edinburgh, EH4 2XU, UK

## Abstract

DNA methylation (DNAm) has been linked to changes in chromatin structure, gene expression and disease. The DNAm level can be affected by genetic variation; although, how this differs by CpG dinucleotide density and genic location of the DNAm site is not well understood. Moreover, the effect of disease causing variants on the DNAm level in a tissue relevant to disease has yet to be fully elucidated. To this end, we investigated the phenotypic profiles, genetic effects and regional genomic heritability for 196080 DNAm sites in healthy colorectum tissue from 132 unrelated Colombian individuals. DNAm sites in regions of low-CpG density were more variable, on average more methylated and were more likely to be significantly heritable when compared with DNAm sites in regions of high-CpG density. DNAm sites located in intergenic regions had a higher mean DNAm level and were more likely to be heritable when compared with DNAm sites in the transcription start site (TSS) of a gene expressed in colon tissue. Within CpG-dense regions, the propensity of the DNAm level to be heritable was lower in the TSS of genes expressed in colon tissue than in the TSS of genes not expressed in colon tissue. In addition, regional genetic variation was associated with variation in local DNAm level no more frequently for DNAm sites within colorectal cancer risk regions than it was for DNAm sites outside such regions. Overall, DNAm sites located in different genomic contexts exhibited distinguishable profiles and may have a different biological function.

## Introduction

Cytosine DNA methylation (DNAm) is a covalent modification of DNA brought about by the addition of a methyl group to the fifth position of the pyrimidine ring of cytosine. In differentiated mammalian cells DNAm occurs almost exclusively at cytosine bases directly upstream of guanine bases (CpG dinucleotide) (1). Importantly, DNAm can be mitotically stable. During embryogenesis the epigenome is erased and reprogrammed, initially prior to blastocyst formation and subsequently in the germ cells (Reviewed in 2–4). In the somatic cells of the developed organism Dnmt1 is the main methyltransferase which targets hemi-methylated DNAm during replication (5). Dnmt1 interacts with a host of proteins at the replication foci including histone deacetylases (6) and histone methyltransferases (7,8) signifying the complex relationship between modification and organization of the chromatin and maintenance of the DNAm level throughout cell division.

Studies have shown that the DNAm level is linked to the gene expression level (9,10). At promoter regions, within a population of individuals average DNAm level and average gene expression level of an associated gene were negatively correlated across genes (9). However, at any given DNAm site, even at a DNAm site within a promoter region, there may be a positive or a negative association between the DNAm site and expression levels of an associated gene across the population of individuals (9). Additionally, the DNAm level has been found to associate with sex and age (10–12) and several environmental factors including early life socioeconomic status and stress (10). Differences in DNAm levels have been observed for DNAm sites in different functional genomic contexts. For instance, DNAm levels tend to decrease towards the TSS of a gene and are relatively high throughout the gene body (1,13,14). Moreover, changes in DNAm levels have been found between DNAm sites located in CpG-dense regions of the genome (CpG islands) and those DNAm sites located outside CpG islands, whether they are close to (CpG island shores and CpG island shelves) or distant from such Islands (13). Furthermore, results from genome-wide association studies, twin studies and studies utilizing mixed linear models indicate that the genotype can affect the level of DNAm (15–22) and that *cis* acting genetic variation can explain a substantial proportion of the phenotypic variation for some DNAm sites (16,17,19,21,22). These studies have been conducted on a limited number of easy to access tissues and report a wide range of heritability estimates for the site-specific DNAm level across the genome.

The average heritability estimate of the site-specific DNAm level varies across tissues and it is likely to be influenced by the method used for estimation and the estimates should be interpreted accordingly. For example, monozygotic and dizygotic twin studies of cells from buccal epithelia and white blood cells estimated the average heritability (${\hat{h}}_{\mathrm{ped}}^{2}$) of all assayed site-specific DNAm levels to be 0.30 and 0.01, respectively(15). Extended families provided an estimate of average ${\hat{h}}_{\mathrm{ped}}^{2}$ for DNAm levels in peripheral blood lymphocytes of 0.20 (20). These studies capture the full extent of the additive genetic heritability. However, sources of shared environmental variance among family members could bias estimates of the additive genetic variance when the environmental variance is un-modelled or difficult to disentangle. The extent of the common environmental effect was tested for peripheral blood lymphocytes (20). The genomic heritability $\left({\hat{h}}_{g}^{2}\right)$, the proportion of the phenotypic variation that can be explained by genetic variation measured by tagging single-nucleotide polymorphism (SNP) on a genotyping array can be estimated using mixed linear models and nominally unrelated individuals (23). However, insufficient linkage disequilibrium between a SNP and the causal variation can mean that not all causal variation, particularly rare causal variation, may be captured by this method. Therefore, ${\hat{h}}_{g}^{2}$ may be biased downwards in relation to the true additive heritability. When using this method, it is straightforward to partition the genetic variation into genomic regions (regional heritability, ${\hat{h}}_{g,r}^{2}$) by simultaneously modelling the effects of SNP within a region of interest (24). Advantages of this method over independently testing the association of each SNP in the genome with a trait (SNP by SNP genome-wide association study) are that small effects within a region maybe compounded into a measureable estimate and effects of rare variants present on a particular haplotype may be captured more effectively (24). Indeed, simulations have shown that in some instances the estimator ${\hat{h}}_{g,r}^{2}$ is more accurate than estimates of heritability obtained from a SNP by SNP genome-wide association study (25). A recent study investigated the effect of the size of the genomic region surrounding a DNAm site on the estimator ${\hat{h}}_{g,r}^{2}$ for DNAm levels measured in the cerebellum, frontal cortex, pons and the temporal cortex for 150 unrelated individuals (22). The authors tested seven region sizes: 10 kb, 50 kb, 100 kb, 500 kb, 1 MB, the local chromosome and the whole genome, and found that using a region size of 50 kb centered around the DNAm site produced the greatest number of significant ${\hat{h}}_{g,r}^{2}$ (22). For instance, depending on the tissue, using the local chromosome or the whole genome reduced the number of significant results to 100 or less whereas using a region of 50 kb produced between 600 and 812 significant results (22). The increase in power that accompanies the optimal region size comes from the smaller number of SNPs modelled within the target region compared with the whole genome. Regions too small will not capture the causative variation. However, because a substantial proportion of the causative genetic variation for the DNAm level is thought to act in *cis*, regions too large will include extraneous SNPs that add noise to the estimator, ${\hat{h}}_{g,r}^{2}$ (22). The average site-specific ${\hat{h}}_{g,r}^{2}$ for regions of ±50 KB around the DNAm site and for DNAm sites with a significant ${\hat{h}}_{g,r}^{2}$ is 0.30 for DNAm levels measured in the cerebellum, frontal cortex, pons and the temporal cortex (22).

The extent to which genetic variation affects variation in the DNAm level may also differ within tissue depending on the functional genomic context of the DNAm sites. For instance, the differences in DNAm levels that have been observed for DNAm sites in different genomic contexts could relate to a difference in the control of the DNAm level. A recent study assessed ${\hat{h}}_{\mathrm{ped}}^{2}$ of site-specific DNAm for DNAm sites located in high- and low-CpG density regions of the genome in peripheral blood lymphocytes (20). This study revealed that estimates in regions of high-CpG density were 0.127 or 0.158, whereas in regions of low-CpG density estimates were greater, 0.235 or 0.223, depending on which probe type (Infinium I or Infinium II) was used to assay the DNAm level. In human brain tissue, the estimator, ${\hat{h}}_{r,g}^{2}$ showed an increased proportion of heritable DNAm sites in regions of the genome with low-CpG density compared with high-CpG density (22). In addition, this same study found a decreased proportion of heritable DNAm sites local to genes up-regulated in a tissue specific manner compared with genes expressed ubiquitously across tissues (22). While these studies have begun to explore the extent of heritability for DNAm sites located in different genomic contexts they have been conducted in a minority of tissues and have considered a limited selection of functional subgroups for DNAm sites.

To build on the work of others who have investigated the phenotypic differences and control of DNAm in different genomic contexts, we assayed 196080 DNAm sites with the Infinium HumanMethylation450K BeadChip (HM450K) in healthy colorectum tissue collected from 132 unrelated Colombian subjects who attended Colonoscopy examination and with diagnosis of hyperplasic polyp, adenoma, *in situ* carcinoma or carcinoma of the rectum or colon. A whole-cell biopsy was taken from the healthy colonic tissue from one of the following locations: ascending, transverse, descending or sigmoid colon, cecum, rectum or region where the sigmoid colon joins the rectum. We grouped the DNAm sites based on location in relation to CpG density, expression status and functional regions of genes. We refer to these groups collectively as contextual groups. Within each contextual group we assessed the profile of the mean site-specific DNAm level where the mean site-specific DNAm level refers to the average DNAm level for a given DNAm site calculated across all the 132 samples. Subsequently, we estimated the effect of local genetic variation on the site-specific DNAm level, using a region size of ±1 MB surrounding the DNAm site following earlier work of others (16,21). We used a regional heritability approach (24) and estimated the proportion of variability in site-specific DNAm levels that is due to local genetic variation, ${\hat{h}}_{r,g}^{2}$. We also contrasted the distribution of ${\hat{h}}_{r,g}^{2}$ for DNAm sites within and outwith known susceptibility loci for colorectal cancer (CRC, OMIM no. 114500). In addition, CRC can manifest in colorectal epithelial cells (26), which are significantly more costly and challenging to extract from the colon than whole-cell biopsies. To establish the extent of the difference in the regulation of DNAm levels in colon epithelial tissue and whole-cell biopsy, we studied the phenotypic profiles and $\;{\hat{h}}_{r,g}^{2}$ for the DNAm level of genes expressed in epithelial cells obtained from laser capture microdissection (LCM) and whole-colon biopsy (WCB).

## Results

### Average DNAm level and relationship to CpG density, genic location and gene expression

**Figure 1.**
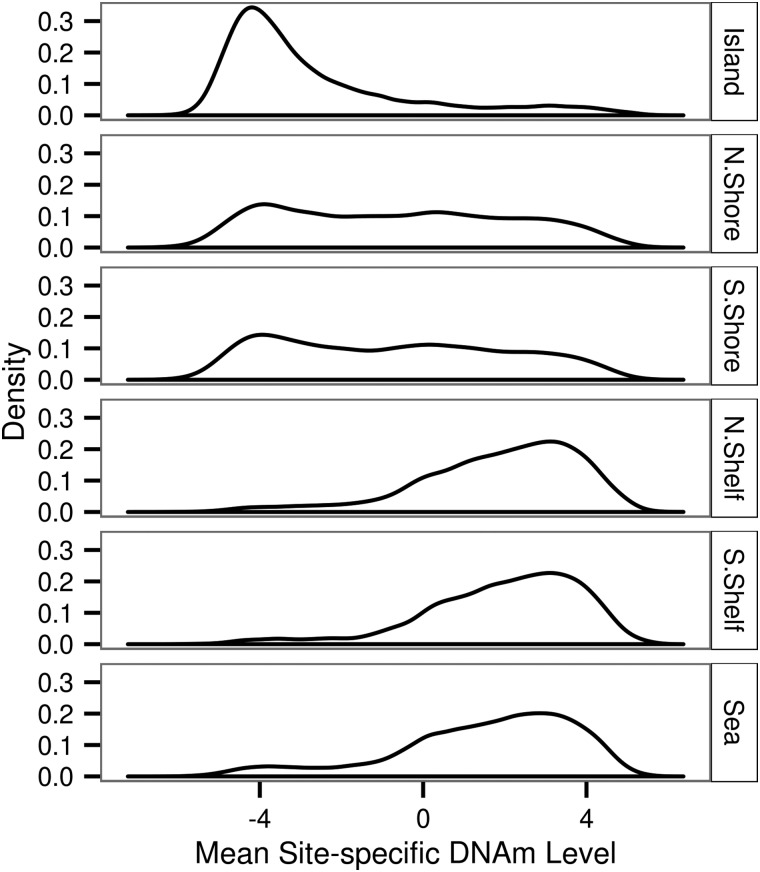
Distribution of mean site-specific DNAm level with respect to CpG density. Methylation levels were measured on the *M*-value scale where a DNAm level of 0 can be interpreted as a 50% methylation level, a DNAm level of <0 and a DNAm level of >0 indicate < and >50% methylation, respectively. The majority of DNAm sites in islands exhibited a low-average methylation level, which was in contrast to the majority of DNAm sites in low-density CG regions (sea) being on average highly methylated.

**Figure 2.**
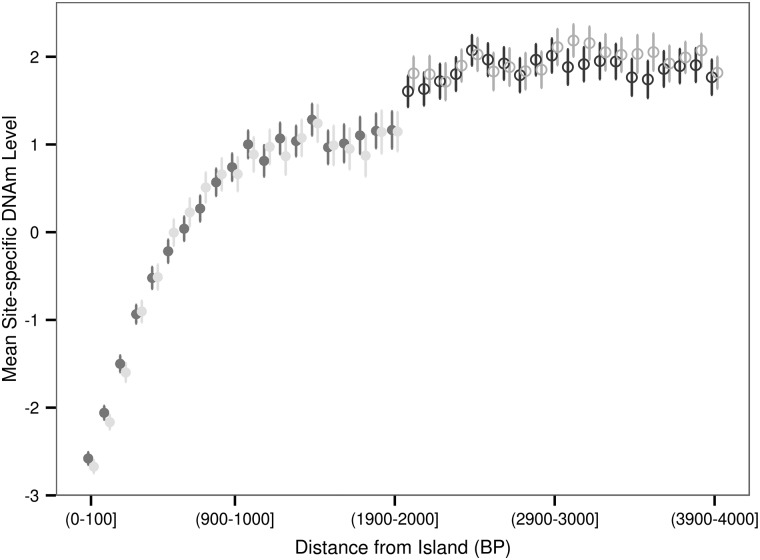
Mean site-specific DNAm level as a function of distance from the edge of the island. The 4000 BP region upstream (North) and downstream (South) of islands was divided into bins of 100 BP. The average of the mean site-specific DNAm levels for DNAm sites residing within each bin is shown as a circle enclosed by a line indicating ±2 standard error of the mean estimate. A shore is up to 2000 BP from an island and a shelf is between 2000 and 4000BP from an island.

In the manifest file for the HM450K array each DNAm site is annotated as being located either within a CpG island, within 2 kb upstream or downstream of an island (north shore and south shore, respectively), within 2–4 kb upstream or downstream of an island (north shelf and south shelf, respectively) or none of the aforementioned categories which we term sea. An island was defined as being composed of one or more adjacent sections of the genome each 500 bp in length with a C and G density >50% and an observed to expected ratio of CpG dinucleotide >0.60 (27). We grouped our 196080 DNAm sites based on aforementioned HM450K array annotation (Table 1). Using the DNAm level values adjusted for gender, age, batch, diagnosis, localization and two genotype principal components we found a substantial difference in the distribution of the average site-specific DNAm levels across contextual groups of varying CpG density (Fig. 1). The average site-specific DNAm level of DNAm sites in islands tended to be much lower than that of DNAm sites located in the sea (mean and median *M*-value was −2.67 and −3.51, and 1.50 and 1.89 for islands and sea, respectively). Additionally, our results showed that the distribution of the average DNAm level for DNAm sites in the north and south shores were similar to one another and more similar to the distribution of the average DNAm level for DNAm sites in islands rather than DNAm sites in the sea (Fig. 1). Conversely, the distribution of the average DNAm level for DNAm sites located in the north and south shelves were similar to one another and were more similar to the distribution observed for DNAm sites located within the sea rather than within islands (Fig. 1). Additionally, we found that within shores the mean site-specific DNAm level is a function of the distance from the edge of the island. The mean site-specific DNAm level increased with distance from the edge of the island in a non-linear fashion (Fig. 2). However, this relationship is not observed for DNAm sites located within shelves (Fig. 2).

**Table 1.** The number of DNAm sites within six regions defined by physical distance from islands

Genomic context with relation to CpG density	Island	North shore	South shore	North shelf	South shelf	Sea	Total
Number of DNAm sites	74274	27405	21158	8323	7503	57417	196080

As described in the body of the manuscript, north and south shores encompass up to 2 KB upstream and downstream of islands respectively. Regions 2–4 KB upstream and downstream of islands were defined, respectively, as north and south shelves (27). Sea is any DNAm site not annotated as being located within an island, shelf or shore in the 450K manifest file.

Unless otherwise specified, the following analyses were based on the two most extreme cases of CpG density: high-CpG density regions (islands) and low-CpG density regions (sea). We tested if the DNAm level of DNAm sites located in the transcription start site (TSS) of genes expressed in WCB was different to those located in the TSS of genes expressed in cells from the colon epithelium collected using LCM. The genes that were expressed in the LCM and the WCB were excluded from the WCB group for this analysis. We found that DNAm sites in the TSS of genes expressed in WCB had an *M*-value that was on average 0.15 >DNAm sites located in the TSS of genes expressed in LCM (mean WCB = −3.32, mean LCM = −3.47, *T*-test *P* = 2.41 × 10^−7^).

Subsequently, we grouped DNAm sites located within the sea or an island into four mutually exclusive sets based on location (i) in a TSS of a gene expressed in WCB (ii) in a TSS of a gene that is not expressed in WCB (iii) in intragenic DNA, where we do not distinguish between genes expressed or not expressed in colon because the methylation level of intragenic DNAm sites has not been correlated with the expression of the surrounding gene or (iv) intergenic DNA (Table 2). Additionally, we choose to use the full set of genes expressed and not expressed in WCB rather than exclusively in colonic epithelial cells because the DNAm level was assayed from WCB. We refer to each of the eight contextual groups individually as: island TSS expressed (within an island and a TSS of a gene expressed in colon), island TSS not expressed (within an island and in a TSS of a gene not expressed in colon), island intragenic (within an island and intragenic), island intergenic (within an island and intergenic), sea TSS expressed (within the sea and the TSS of a gene expressed in colon), sea TSS not expressed (within the sea and the TSS of a gene not expressed in colon), sea intragenic (within the sea and intragenic) and sea intergenic (within the sea and intergenic). Within each of the eight contextual groups, we investigated the distribution of the mean site-specific DNAm level.

**Table 2.** The number of DNAm sites within each of the eight contextual groups

Genomic context	Island	Sea
TSS expressed	13838	2074
TSS not expressed	19052	7603
Intragenic	31356	30106
Intergenic	10028	17634
Total	74274	57417

**Figure 3.**
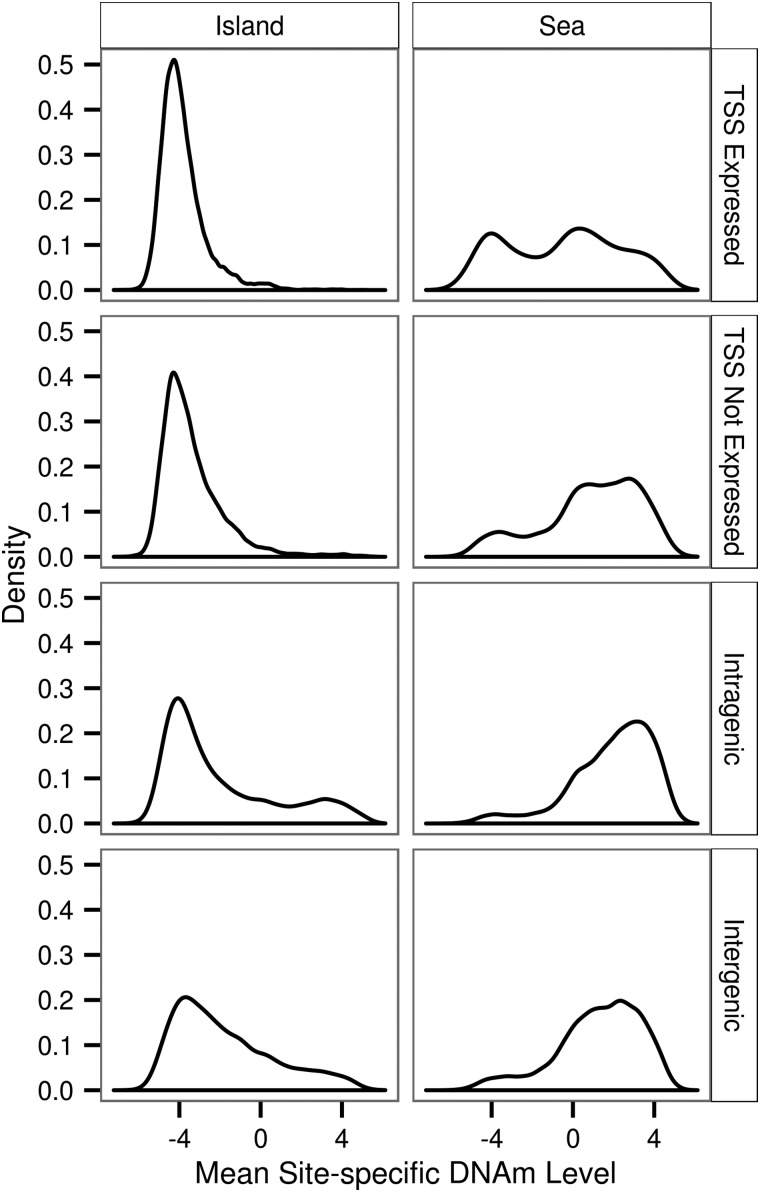
Distribution of mean site-specific DNAm level for eight contextual groups.

**Figure 4.**
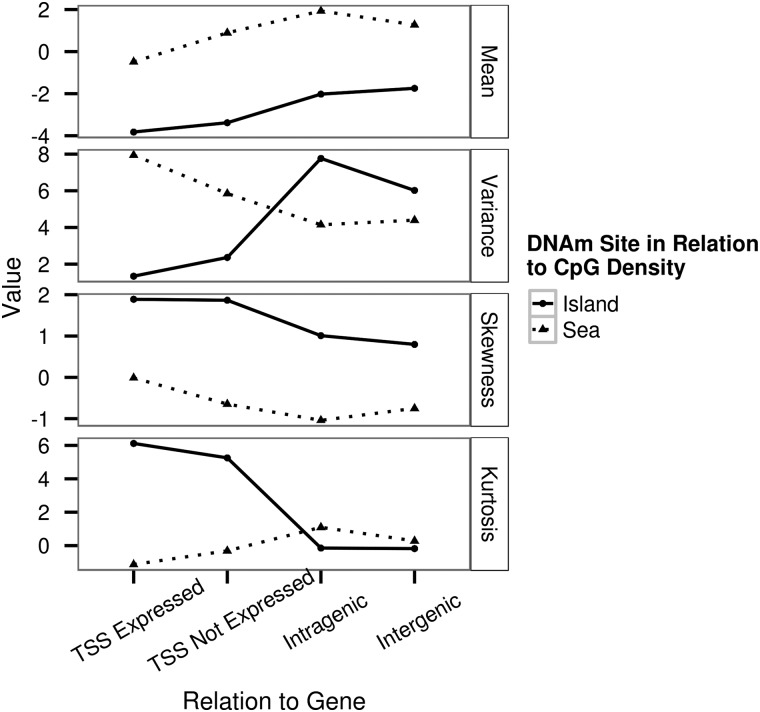
Moments of the distributions of mean site-specific DNAm level for eight contextual groups.

We found a significant difference in the distribution of the mean site-specific DNAm levels between island TSS expressed and island TSS not expressed (Kolmogorov–Smirnov test; *P* < 2.16 × 10^−16^) and between sea TSS expressed and sea TSS not expressed (Kolmogorov–Smirnov test; *P* < 2.16 × 10^−16^) (Fig. 3). We compared the mean of the sea TSS expressed to that of the sea TSS not expressed and we compared the mean of the island TSS expressed to that of island TSS not expressed. These two comparisons were both statistically significant (*T*-test, *P* < 2.16 × 10^−16^, *P* < 2.16 × 10^−16^) and in both cases being located in the TSS of genes not expressed in colon led to an overall greater mean site-specific DNAm level. Additionally, we found that the mean of the distribution of the mean site-specific DNAm level for DNAm sites located in intragenic and intergenic regions was greater than for DNAm sites located in a TSS of a gene (Fig. 4).

**Figure 5.**
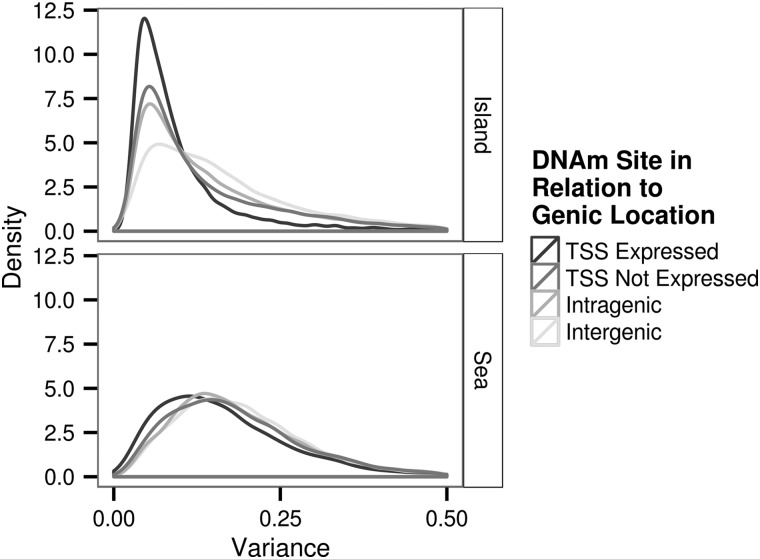
Distribution of the variance of each DNAm site used in analysis (*n* = 196080).

We explored the variation in the DNAm level by investigating the extent to which residual site-specific DNAm level varied across the 132 samples for the full set of 196080 DNAm sites that passed the quality control procedure. The variance of the residual DNAm level ranged between 3.00 × 10^−3^ and 4.91. We observed that on average over all sites the variance of the DNAm levels was significantly higher for DNAm sites located within the sea than for DNAm sites located within an island (mean for sea and island, respectively: 0.198 and 0.152, *P* < 2.2 × 10^−6^ Fig. 5). Moreover, we found the following pattern for the magnitude of mean residual variance both within islands and sea: TSS expressed < TSS not expressed < intragenic < intergenic. Within the islands the difference in mean residual variance was highly significant (*P* < 1.00 × 10^−8^) for all pair-wise comparisons of these categories. Within the sea the difference in mean residual variance was at least moderately significant (*P* < 0.001) for all pair-wise comparisons made except for the comparison of intragenic and intergenic. In this case, the mean residual variance for the sea intragenic and sea intergenic was not significantly different (*P* = 0.240).

### Heritabilities of site-specific DNAm level

**Figure 6.**
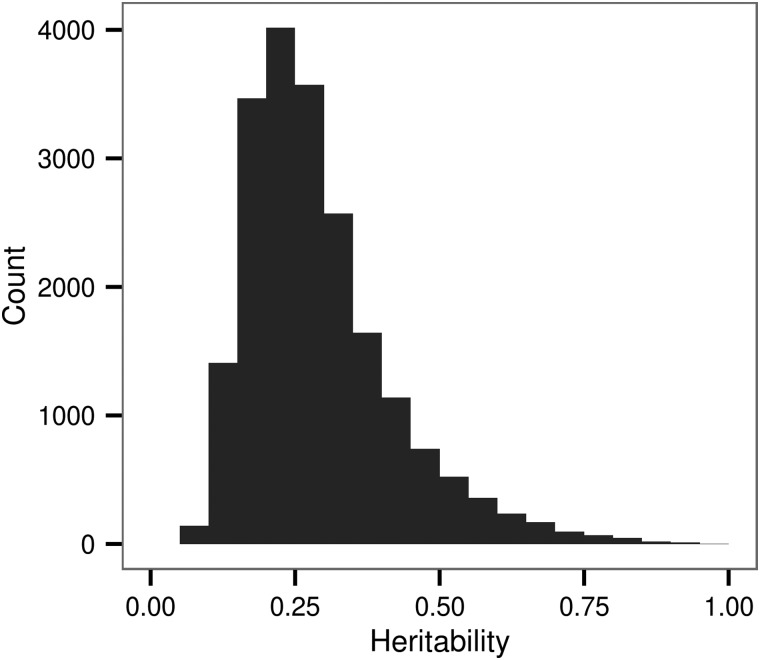
Distribution of the estimated heritability for DNAm sites significantly associated with local genetic variation. Each bar represents a range of 0.05.

**Figure 7.**
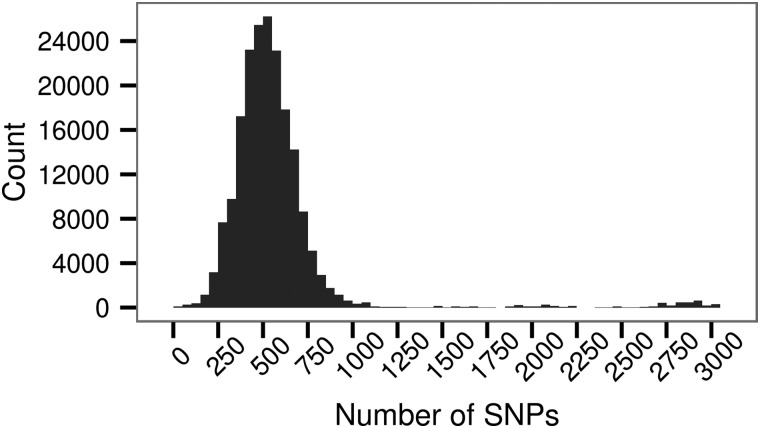
The number of SNPs ±1 MB surrounding a DNAm site.

We found that at 20239 DNAm sites, 10.32% of the 196080 tested, SNPs within 1 MB explained a significant proportion of the variation in the methylation level (nominal *P* < 0.05). The percentage of heritable DNAm sites exceeds that expected from a false-positive rate of 0.05 under the null hypothesis that the DNAm level is not associated with local genetic variation. For significantly heritable loci, the proportion of the variance in DNAm under local genetic control ranged between 0.06 and 0.99 with a mean of 0.29 and median of 0.26 (Fig. 6). We found that the numbers of SNPs in the local region (Fig. 7) explain a minute but significant proportion of the variance in heritability estimates for the DNAm sites with a significant heritability (univariate linear regression: *R*^2^ = 0.005, slope = 2.372 × 10^−5^, *P* < 2.2 × 10^−16^). For instance, considering the range of the number of SNPs within a region, at the first decile (304 SNPs) and ninth decile (2733 SNPs), we expect a respective 7.2 × 10^−3^ and 6.5 × 10^−2^ increase in the heritability for the DNAm sites found to be significantly associated with local genetic variation. In addition, we found that the variance of the residual DNAm level at each DNAm site explained a proportion of 1.93 × 10^−2^ of the variance in the heritability estimate (univariate linear regression: *P* < 2.2 × 10^−16^). An analysis of variance indicated that genomic context explained a significant proportion (*P* < 2.2 × 10^−16^) of the variability in the heritability estimate after accounting for residual variance.

### Heritability of DNAm level in genes expressed in whole-colorectal biopsies and colorectal epithelial

**Figure 8.**
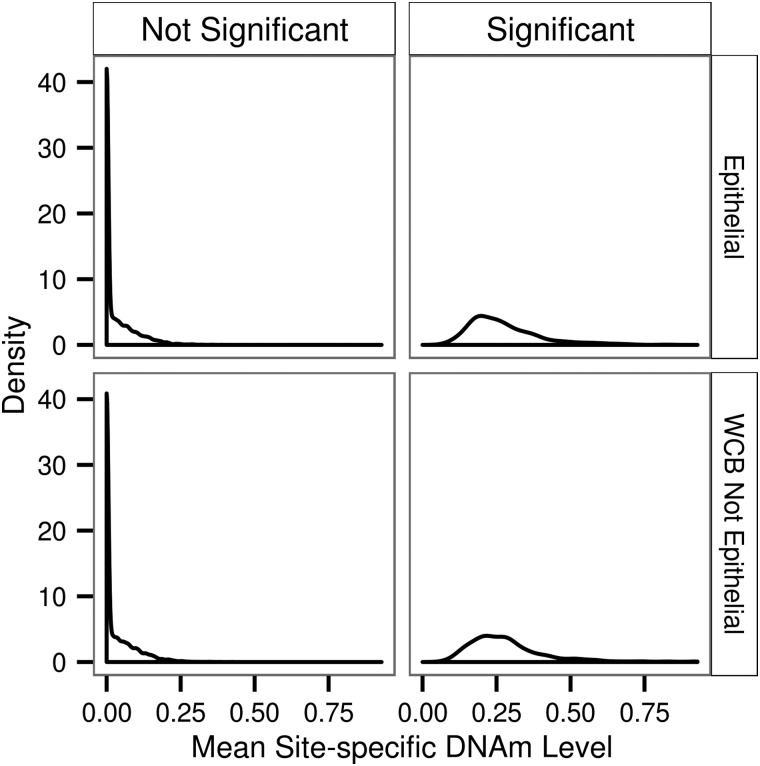
Distribution of mean-site specific heritability for DNAm level in genes expressed in whole colorectal biopsies and colon epithelial cells. DNAm sites have a significant heritability if *P* < 0.05. Genes expressed in both the epithelial and WCB were removed from the WCB group for this analysis.

We investigated the heritability of the site-specific DNAm level for genes expressed in LCM and in WCB excluding those expressed in LCM (Fig. 8). The difference between the average of the mean site-specific DNAm levels for the significantly heritable DNAm sites within the two groups was not significant (mean LCM = 0.272, mean WCB = 0.283, *T*-test *P* = P = 0.151). Additionally, the proportion of significantly heritable DNAm sites in the LCM and WCB groups was 0.0723 and 0.0734, respectively, and was not significantly different from one another (*P* = 0.0834).

### Heritability of DNAm sites in whole-colorectal biopsies by genomic context

The proportion of sites with a significant heritability was higher in the sea than in islands (*P* < 2.20 × 10^−16^, Table 3). This result was driven by the difference between DNAm sites located within the TSS of a gene or in intragenic regions. The proportion of heritable sites is 1.54 times higher for DNAm located in the TSS of the sea than in the TSS of an island (*P* < 2.20 × 10^−16^); additionally, the proportion of heritable DNAm sites is 1.13 times higher for DNAm sites located in the sea intragenic than the island intragenic contextual group (*P* = 1.47 × 10^−6^). There was no significant difference in the proportion of heritable DNAm sites located in intergenic regions when comparing between the sea and island contextual groups (*P* = 0.125).

**Table 3.** Proportion of heritable DNAm sites and the corresponding mean heritability

	Island		Sea	
	Proportion heritable	Mean, *h*^2^	Proportion heritable	Mean, *h*^2^
TSS expressed	0.066	0.275	0.117	0.288
TSS not expressed	0.084	0.281	0.117	0.293
Intragenic	0.089	0.283	0.100	0.290
Intergenic	0.129	0.308	0.123	0.303
Total	0.089	0.286	0.110	0.295

Overall there was a higher proportion of heritable DNAm sites located in the sea compared with islands. Additionally, there was a higher proportion of heritable DNAm sites located in intergenic regions compared with regions containing a TSS and intragenic regions. The average heritability estimates were similar across the contextual groups.

The proportion of heritable DNAm sites was lower for sea intragenic than the other three sea contextual groups. This difference was highly significant for the comparison of sea intragenic and sea intergenic (*P* = 1.55 × 10^−14^) and for the comparison of sea intragenic and sea TSS not expressed (*P* = 2.65 × 10^−5^). The difference was significant at a nominal threshold (*P* < 0.05) for the sea intragenic and sea TSS expressed comparison (*P* = 1.52 × 10^−2^). The proportion of heritable DNAm sites was significantly different for all comparisons made within the island contextual groups. This difference was highly significant (*P* < 1.00 × 10^−8^) except for the island TSS not expressed and island intragenic comparison (*P* = 4.34 × 10^−2^). Within the island contextual groups the proportion of heritable DNAm sites was as follows: intergenic > intragenic > TSS not expressed > TSS expressed.

**Figure 9.**
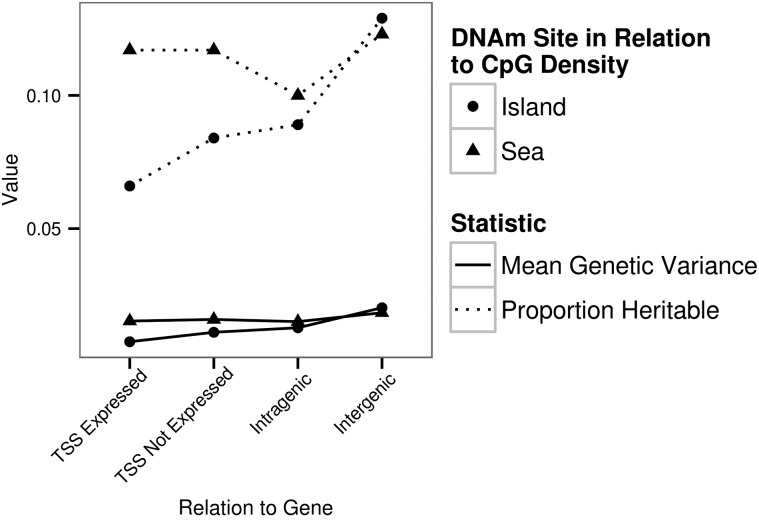
The mean genetic variance and the proportion of heritable DNAm sites for the eight contextual groups. The *x*-axis value represents the proportion of heritable DNAm sites within each contextual group and the average genetic variance for each contextual group.

The proportion of heritable DNAm sites for each contextual group followed a similar pattern to the mean local genetic variance for each contextual group (Fig. 9). Across each four sea and island contextual groups the proportion of heritable DNAm sites was correlated with the mean local genetic variance (Pearson's correlation: *r* = 0.700 and *r* = 0.999 for sea and island, respectively).

The average ${\hat{h}}_{r,g}^{2}$ for significantly heritable DNAm sites located within each of the genomic contexts was similar (Table 3). DNAm sites significantly associated with local genomic variation and located within an island were on average 0.9% less heritable than DNAm sites located in the sea.

### SNP by SNP GWAS for the DNAm level at 196080 DNAm sites

**Figure 10.**
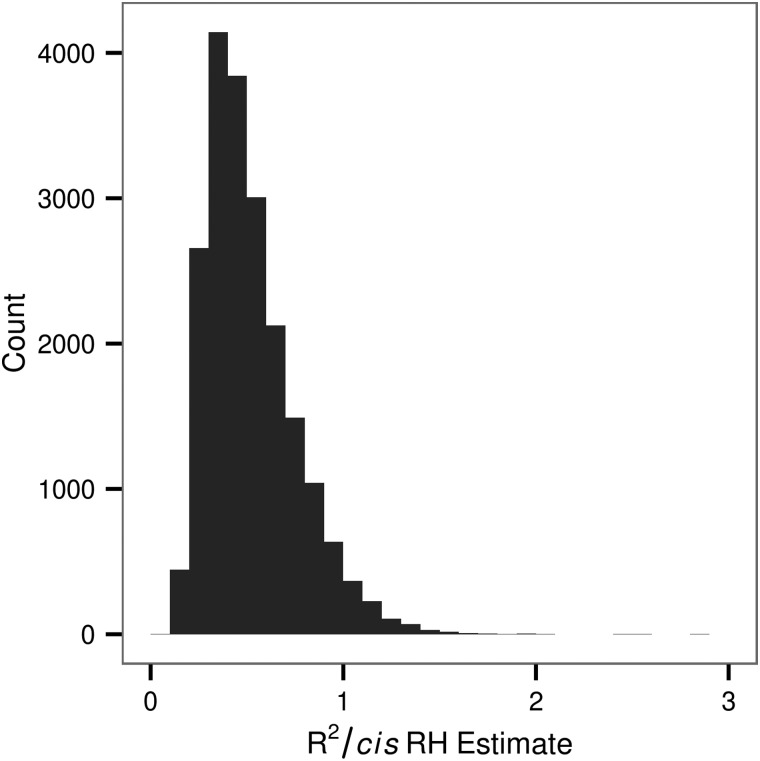
Proportion of the regional heritability that can be explained by the top SNP association. The variance explained by a SNP (*R*^2^) divided by the regional heritability estimate for DNAm sites with a regional heritability estimate significant at *P* < 0.05. Only the most significant SNP within the local region was considered. In 4.2% of cases the SNP explained more variance in the DNAm level than did the region.

We conducted a GWAS for each of the 196080 DNAm sites to localize causal variation within each of the *cis* regions and to determine if there was evidence for genetic effects on the DNAm level in *trans*. We found enrichment for *cis* and *trans* genetic effects on the DNAm level beyond what would be expected by chance assuming the test for the association of each SNP and DNAm site are independent (Table 4). DNAm sites with at least one significant *cis* SNP association were found on average to have a minimum of two associated-*cis* SNPs, where the average number depended on the threshold specified (Table 4). Each DNAm site with a significant RH estimate (*P* < 0.05, *n* = 20239) was paired with the SNP to which it was most significantly associated with in *cis*. We calculated the proportion of the regional heritability estimate explained by variation of the SNP for each pair (Fig. 10). In the majority of cases (95.8%) the region explained equal or more variance in the DNAm levels than the single most significant SNP (Fig. 10).

**Table 4.** Significant associations from SNP by SNP GWAS for 196080 DNAm sites

Threshold	*Cis*			*Trans*		
	Count	Count expected	Count DNAm sites	Count	Count expected	Count DNAm sites
5 × 10^−2^	6 027 276	5 572 851	195 792			
5 × 10^−4^	184 978	55 729	55 532			
5 × 10^−8^	17 712	5.57	4050	1 519 534	6690	72544
5 × 10^−12^	9,495	5.57 × 10^−4^	1412	236 577	6.69 × 10^−1^	16474
5 × 10^−20^	1037	5.57 × 10^−12^	343	23 072	6.69 × 10^−9^	1786
5 × 10^−40^	18	5.57 × 10^−32^	8	706	6.69 × 10^−29^	47
Max *R*^2^	0.884			0.8717		
Min *P*-value	1.40 × 10^−62^			1.96 × 10^−58^		
Total SNPs Tested	111558037			1.34 × 10^11^		

The total number of significant associations (count) and number of expected significant associations based on the specified threshold and the total number of SNPs tested (count expected) is reported. The number of DNAm sites with at least one significant association is given (count DNAm sites). SNPs were binned with respect to distance from the DNAm site (*cis* ≤ ±1 MB and *trans* > ±1 MB).

### Heritability of DNAm sites in whole-colorectal biopsies with respect to loci associated with colorectal cancer

An extensive number of genetic variants have been found to associate with complex disease including CRC (28). However, in the majority of cases how the identified genetic risk variants act to increase disease susceptibility is unknown. Genetic variation could increase risk to disease by mediating changes in DNAm levels in healthy tissue. If an association between a susceptibility SNP and DNAm level in healthy tissue is obtained, one possibility is that the variation in the DNAm level interacts with additional variables, such as environmental factors, to subsequently lead to disease. Therefore, we sought to determine if variation in DNAm levels in healthy colon tissue was associated with genetic variation that incurs susceptibility to CRC. To this end, a total of 83 unique autosomal SNPs identified as associating with CRC were downloaded from the NHGRI GWAS catalogue (28). We defined a region of ±1 MB surrounding each SNP associated with CRC as a risk region. A total of 10469 DNAm sites were located within a defined risk region and due to our definition of a risk region the calculation of ${\hat{h}}_{r,g}^{2}$ included the effects at the position of the risk SNP. Indeed, we found that an equal proportion (0.103) of DNAm sites were heritable within and outwith risk regions. The average heritability was also similar for DNAm sites located within and outwith risk regions (within = 0.290, outwith = 0.292).

In conjunction, a recent study (29) found that DNAm levels of two DNAm sites measured in healthy colorectal tissue, cg15193198 and cg24112000, were associated (FDR < 0.05) with the local CRC risk variant rs4925386 located on chromosome 20 at 60921044 base pairs. In our study, both cg15193198 and cg24112000 were significantly heritable both when including rs4925386 in the calculation of the genetic relationships and when excluding rs4925386 (Table 5). We determined that rs4925386 explained 92 and 55% of the ${\hat{h}}_{r,g}^{2}$ estimate for cg15193198 and cg24112000, respectively, and ${\hat{h}}_{r,g}^{2}$ was still significant for cg24112000 when fitting rs4925386 as a fixed effect (Table 5).

**Table 5.** Effects of rs4925386 and local genetic variation on cg15193198 and cg24112000

	${\hat{h}}_{r,g}^{2}$	${\hat{h}}_{r,g}^{2}$, *P*-value	${\hat{h}}_{r,g}^{2}$ full	${\hat{h}}_{r,g}^{2}$ full *P*-value	${\hat{h}}_{r,g}^{2}$ reduced	${\hat{h}}_{r,g}^{2}$ reduced, *P*-value	SNP effect	SNP effect, SE
cg15193198	0.307	1.66 × 10^−3^	0.025	0.411	0.301	2.19 × 10^−3^	−0.438	0.079
cg24112000	0.625	5.51 × 10^−12^	0.281	1.44 × 10^−2^	0.629	8.47 × 10^−12^	−0.576	0.090

The regional heritability estimate (${\hat{h}}_{r,g}^{2})$ for cg15193198 and cg24112000 including rs4925386. The estimate for the full model (${\hat{h}}_{r,g}^{2}$ full) and reduced model (${\hat{h}}_{r,g}^{2}$ reduced) were calculated from all SNPs ≤ ±1 MB of the DNAm site excluding rs4925386. The full model included fitting the genotypes at rs4925386 as a fixed effect. The effect of rs4925386 on the DNAm level is reported as the addition of a single copy of the minor allele, adenine.

## Discussion

DNAm sites located in different genomic contexts with respect to CpG density and genic location exhibit unique profiles in the human colorectum. We have shown that the average DNAm level is related to CpG density and genic location. The relationship of the DNAm level with CpG density has been observed for the DNAm level measured at promoters in peripheral blood mononuclear cells and fibroblasts (10,30) and concurs with that found by a recent study profiling DNAm level in 17 somatic tissues (13). In addition, we find that specifically within shores that there is a shift in the average DNAm level from predominantly unmethylated to methylated as distance increases from the edge of the CpG dense islands. This change in the DNAm level is suggestive of a transitional zone at the edge of islands captured by the definition of shore. Overall, the lower and less variable average site-specific DNAm level of DNAm sites located in islands compared with sea is consistent with the traditional view that CpG-dense regions of the genome persist due to low methylation and a reduced rate of spontaneous deamination and transition that is typically higher for methylated CpG dinucleotides (31). Our finding that irrespective of CpG density, the DNAm level was lower in the TSS than within intergenic or intragenic regions concurs with what has been observed in H1 embyronic cells where the DNAm level has been shown to decrease between the promoter and 5′UTR region before increasing through the gene body and into the 3′UTR (1). The level of DNAm has also been shown to be greater in intragenic and intergenic regions compared with promoter regions in human brain frontal cortex grey matter (32). Lower variation of the DNAm level within TSS compared with the intragenic and intergenic regions and within islands supports the idea that DNAm in CpG-dense regions of the genome and in TSS targets housekeeping genes (30,33). Housekeeping genes are essential for normal cell maintenance and thus expression of these genes may be tightly regulated and this could be reflected by the low level and low variation of the site-specific DNAm level in these regions. Additionally, DNAm sites located in the TSS of a gene not expressed in WCB were on average more methylated than DNAm sites located in the TSS of a gene that was expressed in WCB. This result is suggestive of an overall inverse correlation between mean gene expression and mean DNAm level which has previously been observed (9).

We have assessed, on a genome-wide scale, the local heritability of the site-specific DNAm level in normal WCB using unrelated Colombian individuals. A total of 10.32% of DNAm sites in WCB were significantly affected by local genetic variation. The mean ${\hat{h}}_{r,g}^{2}$ for the heritable sites was 0.29 but the estimates vary substantially with some DNAm sites exhibiting a low heritability and some DNAm sites exhibiting heritability close to one. The implication is that the DNAm level can be inherited through the germ-line. These results are consistent with previous estimates of the number of DNAm sites and gene expression probes across the genome affected by local genetic variation and with the wide range of heritability estimates reported for levels of DNAm and gene expression (16,22,34–36). Indeed, we found that that there were an increased proportion of significantly heritable DNAm sites located in the sea compared with islands. Overall, our finding that heritable DNAm sites were enriched for location outside of islands is in accordance with what is observed in human brain tissue (22). We hypothesize that the substantial difference in the mean estimates of ${\hat{h}}_{\mathrm{ped}}^{2}$ for DNAm sites located in islands and in sea obtained in peripheral blood lymphocytes (20) and outlined in the background section of this paper may have resulted from (i) the inclusion of all DNAm sites rather than just those with a significant heritability estimate (ii) the use of the pedigree to estimate the contribution of the whole genome to phenotypic variance and/or (iii) bias due to un-modelled sources of environmental variation. In conjunction, CG content ±5 KB of a TSS was inversely associated with the (genome-wide) heritability of gene expression measured in peripheral blood from 1444 twin pairs (36).

The highest proportions of significantly heritable DNAm sites were located in intergenic regions as opposed to within the TSS of a gene or intragenic regions. Moreover, for DNAm sites within islands, being located in the TSS of a gene expressed in colon tissue led to a significantly lower probability of being heritable compared with being located within the TSS of a gene not expressed in colon tissue. However, this pattern was not observed within the sea. This is suggestive of forces acting in a different manner at TSS within islands compared with at TSS within sea to maintain DNAm levels. Overall, the proportion of heritable DNAm sites was correlated with the average estimated genetic variance. A similar observation has been made for gene expression in Epstein-Barr virus transformed LCLs (37). In this case, the lower proportion of heritable DNAm sites observed for a contextual group(s) such as islands compared with other contextual group(s) such as the sea can in part be explained by a reduction in the measured genetic variance. Lower genetic variation could result in lower power to capture the true causative loci or it could be indicative of lower causal variation due to selective constraints.

The DNAm level was slightly higher at the TSS of genes expressed in WCB and not LCM compared with those expressed in LCM. This result is consistent with the WCB samples being enriched for epithelial cells and a negative correlation between gene expression and the DNAm level. However, the overall heritability of the DNAm level at the TSS of genes expressed in WCB and not LCM compared with those expressed in LCM was similar. One possible explanation for these results is that in healthy colonic tissue the genes expressed in the colon epithelium are regulated by the DNAm level in a similar fashion to the genes expressed in the WCB.

Finally, we have shown that genetic variants in genomic risk regions for CRC can affect the DNAm level in healthy colon tissue and that overall DNAm sites within a risk region have similar overall heritability to DNAm sites outwith an identified risk region. In conjunction, we have replicated the previous finding that the CRC risk SNP rs4925386 effects DNAm level at cg15193198 and cg24112000.

We showed that when rs4925386 is excluded the regional genetic variation sufficiently captures the causal variation in the DNAm level tagged by rs4925386. Moreover, rs4925386 alone does not capture all the genetic variance contributing to variation of cg24112000 and cg15193198 that is captured by the regional heritability approach. This final result highlights the advantage of the regional heritability approach to capture the genetic effects on the phenotype, in this case DNAm level.

We have identified individual SNPs outwith ±1 MB a DNAm site which affect the DNAm level. However, studies of larger sample size are required to estimate the combined long-range effects (polygenic effect) of genetic variants on the DNAm level with nominally unrelated individuals using the regional heritability method. This is because variance in the extent of identify by descent between nominally unrelated individuals is lower across the genome as a whole than it is within a small genomic region. However, in accordance with (22), we have shown that a small sample of nominally unrelated individuals can be used to estimate the genetic contribution of a genomic region to variation in the DNAm level.

We have identified a subset of DNAm sites genome-wide and measured in healthy colon tissue that are influenced by the local genetic variation. Therefore, we have contributed to understanding healthy genetically influenced variation in the DNAm level in colon tissue. A number of the DNAm sites which we report as heritable are located within CRC risk loci and thus have the potential to mediate genetic susceptibility to CRC. We expect further studies will focus on exploring a role for these DNAm sites in disease aetiology.

## Materials and Methods

### Samples

A total of 144 samples from normal colorectal tissue were obtained from Colombian patients diagnosed with hyperplastic polyps, adenomas or adenocarcinoma of the colorectum. The study had ethical approval from the Ethics Board of The National Cancer Institute of Colombia, and participants gave informed written consent.

Similarly, 12 people undergoing colonoscopic examination at General University Hospital of Elche (Spain) but without adenocarcinoma or adenomas of the colorectum provided tissue samples from the colon. Written informed consent for inclusion in the study was obtained from every participating individual. The study was approved by the ethics committees of the General University Hospital of Elche.

### Phenotype QC

We assayed 144 samples for the DNAm level at 485512 DNAm sites using the HM450K array.

Within each colorectal tissue sample the two intensity values that correspond to the number of methylated and unmethylated copies of a DNAm were corrected for any variation that arose from non-specific binding. This background correction was applied by subtracting the median fluorescence measured by the control probes from the intensity values treating intensities measured in the two colour channels separately and using the bioconductor package, ‘lumi’ (38). Subsequently, nine samples for which the assaying process failed were identified. These samples had a low-average intensity value (<2500) measured in either or both of the colour channels and were removed. We examined the percentage of probes that were not detected above background levels of variation (*P* = 0.01) for each sample and found that no samples exceeded our threshold of 5% for exclusion. Two samples where the recorded sex of the individual did not match the sex estimated from the levels of DNAm measured on the X chromosome were removed. A total of 280 469 DNAm probes were removed because they contained a SNP within the target sequence or at the site of single-base extension or they were deemed cross-reactive based on the work of (39) and the information provided in the HM450K manifest file. Additionally, 354 probes were removed because they were not detected above background levels of variation for >5% of samples (*P* < 0.01). This resulted in 133 samples and 196080 autosomal DNAm probes left for downstream analysis. Colour bias was taken into account by comparing for all samples the within sample distribution of total intensities measured by the type I probes in the green channel to those measured in the red channel. A quantile normalization adjustment was applied within the bioconductor package, ‘lumi’ (38), so that the intensity values measured in the two colour channels followed a similar distribution across and within individuals. We also applied a correction to account for technical variation due to the probe design type using the BMIQ algorithm (40).

We conducted analyses and reported levels of DNAm using the *M*-value scale. This scale reduces the dependence between the variance and mean of the site-specific DNAm level that is observed on the beta scale (41). *M*-values are a logit transformation of the *β*-values and an *M*-value of 0 equates to a 50% level of methylation whereas a positive and a negative *M*-value relates to a greater and less than 50% methylation level, respectively.

### Genotype QC

We genotyped 468 samples at 958178 SNPs genome-wide using the Illumina HumanOmniExpress Exome Chip. We followed a standard quality control procedure (reviewed in 42) using Plink (43). Four samples for which >5% of SNPs did not genotype were excluded. Based on the application of four successive filters, SNPs were removed if (i) they failed to type in >5% of samples or (ii) if they were out of Hardy–Weinberg equilibrium (*P* < 0.0001) or (iii) if they had a MAF of <0.01 or (iv) if the rate of genotype failure was significantly different in cases and controls (*P* < 0.00001). This procedure left a total of 682945 autosomal SNPs for analysis. Additionally, the inbreeding coefficient for each sample was calculated from SNPs along the X chromosome. This analysis revealed 3 samples recorded as female that were more inbred than expected (*F* > 0.98) and 10 samples recorded as male that were less inbred than expected (*F* < 0.2). These samples were removed from subsequent analysis due to an assumed discrepancy between the recorded and observed identity. This left 132 samples with quality genotype information that overlapped with the samples assayed for the DNAm level and which passed the DNAm level quality control procedures.

### Identification of genes expressed in colon tissue

Genes expressed in general colon tissue and specifically in colon epithelial cells were identified based on the analysis of normal tissue from biopsies of 12 people undergoing colonoscopic examination at General University Hospital of Elche (Spain). In order to separate epithelial specific expression, tissue samples were sliced with alternate slices assigned to the *whole-tissue* and *epithelial* conditions. In the whole-tissue condition combined slices for each individual were assayed for gene expression. In the epithelial condition we pooled epithelial cells, isolated using Laser Capture Microdisection (MMI CellCutPlus), from each slice for each individual and mRNA from the slices was amplified prior to being assayed for gene expression. The gene expression assay on the 24 samples was performed using the HumanHT-12 Expression BeadChip. Quality control indicated failure of four samples (one in the whole tissue and three in the epithelial condition) which were removed from subsequent analysis. We then identified for each condition mRNA probes which were detected above background (*P* < 0.01) in >80% of samples, i.e. 9 or more of 11 samples and 8 or more of 9 samples for the whole-tissue and epithelial conditions, respectively. This yielded 9223 probes in the whole tissue and 4071 probes in the epithelial conditions. Probes were mapped to genes using the Illumina provided manifest file for the HumanHT-12 Expression BeadChip platform, yielding a list of 8114 genes expressed in general colon tissue and 3754 genes expressed in epithelium. As expected a majority of genes identified in the epithelial condition were also detected in the whole tissue, which contains both the epithelial and other cells, with only 10 specific to the epithelial condition. This supports the view that the genes identified form subset of genes enriched for epithelial specific expression.

### Location of a DNAm site with respect to a gene

A DNAm site was considered to be located within the TSS of a gene expressed in colon tissue if in the manifest file the site was recorded as being located within 200 bp upstream of the TSS (TSS200) or, within 200–1500 bp upstream of the TSS (TSS1500) of a gene in our list of expressed genes. All other DNAm sites located within the TSS200 or TSS1500 region of a gene were considered as being located within a gene not expressed in colon tissue. Intragenic DNAm sites were those documented in the manifest file as located within the 5′UTR, first exon, gene body or 3′UTR. Intergenic DNAm sites were those not documented as residing within a gene. We applied successive filters in the aforementioned order so that each DNAm site fit into one of the four mutually exclusive categories.

### Statistical analysis

A WCB of healthy tissue was obtained from the ascending, transverse, descending or sigmoid colon, cecum, rectum or region where the sigmoid colon joins the rectum from Colombian subjects who attended Colonoscopy examination and with diagnosis of hyperplasic polyp, adenoma, *in situ* carcinoma or carcinoma of the rectum or colon. In our analyses, we included both diagnosis and biopsy location as explanatory variables. Biological differences between the right (proximal) and left (distal) colon have been identified and they include the tissue of developmental origin and manifestation of CRC (44). Therefore, we used the WCB location to define a new variable that indicated the location of the WCB with respect to left and right colon. The right colon included the cecum, ascending and transverse colon. The left colon included the descending and sigmoid colon, sigmoid-rectum union and the rectum. This variable was used as the explanatory variable to adjust for the effects of WCB location. We also accounted for sex, age and batch (plate) in our analyses following the work of others who have shown that these variables can affect the DNAm level (10,11,12,16). In addition, we fitted two genotype principal components to account for any stratification within our population sample. Results were practically identical when the analyses were done adjusting for sex and age.

The components of variance were estimated by fitting a mixed linear model using restricted maximum likelihood and the publically available software: REACTA (45). Consider *y* a vector of measurements of DNAm level across all samples (*n*) for a single DNAm site, *β* a vector measuring the effects sex and age and X a design matrix mapping the appropriate explanatory variable to each sample. Then with *W* a matrix of standardized SNP genotypes from each sample and assuming a vector of SNP effects, $u∼N(0,I\sigma_{u}^{2})$ with *I* a diagonal matrix and the random error $\epsilon ∼N(0,I\sigma_{\epsilon }^{2})$ the model is defined as:

y=Xβ+Wu+ϵ

The heritability attributable to SNPs local to the DNAm site can be estimated from the following equation:

$$h^{2}\frac{\sigma_{g}^{2}}{\sigma_{g}^{2}+\sigma_{\epsilon }^{2}}$$

where $\sigma_{g}^{2}=N\sigma_{u}^{2}$ and N is the number of SNPs, and $A=WW^{\prime }/N$ is a matrix of genetically derived relationships calculated from *N* SNPs for *n* samples. The formula used for calculating the pair-wise relationships from the SNP information can be found in VanRaden (46). Using a 1 MB window either side of the DNAm we find that a total of between 1 and 3037 SNPs are included in the analysis (Fig. 7). The null hypothesis that the heritability estimate was not significantly different from zero was tested with a likelihood-ratio test distributed as 50:50 mixture of *χ*^2^distributions with 0 and 1 degrees of freedom (47). If *P* < 0.05 we rejected the null hypothesis and concluded that the DNAm site was heritable.

The SNP by SNP GWAS was conducted on the residual DNAm level for each of 196080 DNAm sites using PLINK version 1.90 (43) and the --assoc command.

### Significance testing of proportions

To test if two proportions are significantly different from one another we use the prop.test function in R (48). In brief, this function assumes that the two sample sizes are sufficiently large so that the distribution of the first proportion minus the second proportion is Gaussian. We apply a two-tailed test because we do not have prior expectation of the relative magnitudes of the two proportions being tested.

## Funding

This work was supported by National Cancer Institute of Colombia, Cancer Research UK (C12229/A13154) and The Roslin Institute Strategic Grant funding from the Biotechnology and Biological Sciences Research Council (BB/J004235/1). A.T. and C.S.H. also acknowledges funding from the Medical Research Council Human Genetics Unit. Funding to pay Open Access charges was provided by the Biotechnology and Biological Sciences Research Council (BB/J004235/1).
